# Functional Properties of Sonochemically Synthesized Zinc Oxide Nanoparticles and Cotton Composites

**DOI:** 10.3390/nano10091661

**Published:** 2020-08-25

**Authors:** Muhammad Tayyab Noman, Michal Petrů

**Affiliations:** Department of Machinery Construction, Institute for Nanomaterials, Advanced Technologies and Innovation (CXI), Technical University of Liberec, Studentská 1402/2, 461 17 Liberec 1, Czech Republic; michal.petru@tul.cz

**Keywords:** C-nZnO composites, nZnO, functional properties, leaching durability, washing stability

## Abstract

In this study, zinc oxide nanoparticles (nZnO) were synthesized, deposited, and successfully used for surface modification of cotton to enhance antimicrobial properties. An in situ ultrasonic acoustic method was applied to anchor nZnO on cotton. The results of scanning electron microscopy, energy dispersive X-ray spectroscopy, and X-ray diffraction confirmed the presence of nZnO on cotton. A homogenous distribution of nZnO with an average particle size 27.4 nm was found during the analysis of results. Antimicrobial performance of cotton-nZnO (C-nZnO) composites was evaluated against Gram-negative and Gram-positive microbes. The deposited amount of nZnO on C-nZnO composites was determined by volumetric titration through inductive couple plasma atomic emission spectroscopy. C-nZnO composites showed excellent antimicrobial performance especially against both *Staphylococcus aureus* (Gram-positive) and *Escherichia coli*. The durability and stability of C-nZnO composites were tested against leaching and washing. No significant fluctuation was found on deposited amount of nZnO before and after washing test for optimized sample. The results demonstrate that synthesized C-nZnO composite samples can be used as an alternative for antimicrobial bandages.

## 1. Introduction

The textile sector opts systematic changes, similar to other industries, to develop new and robust products which fulfill the customer expectations. These changes enhance and accelerate the use of textiles in different industries. At present time, there are plenty of textile products present in the market based on their functionality and the most popular functional textile products with significant antimicrobial and self-cleaning properties are used in medical, materials, and polymer industries [1,2,3,4,5]. The high popularity and demand of functional textiles in medical field is due to the increasing resistance of microbes towards drugs. The materials used for antimicrobial finishes in textiles not only prevents the reproduction of microorganisms i.e., bacteria, fungi, and pathogens, but also control the pathogens spreading [6]. Therefore, these materials are successfully used in the production of disposable medical textiles, i.e., surgical gloves, wound dressings, caps, protective aprons, surgical curtains, as well as reusable medical textiles, i.e., towels, bed sheets, and work clothes. Another use of these materials is in sports clothes for long-lasting freshness. Chitosan [7,8,9,10], ammonium salts [11,12,13,14], triclosan [15,16], and metal oxide nanoparticles [17,18,19,20,21] are the examples of most common materials used for functional textile products. These agents can also be classified into organic and inorganic compounds. Zinc oxide (ZnO) is one of the materials belongs to metal oxide family with excellent UV absorption, photocatalytic activity, antimicrobial and self-cleaning properties, and photo-oxidizing ability against chemical and biological species [22].

The miracles of nanoscale synthesis have made it possible in the textile industry to fabricate a new generation of antimicrobial finish on fabric surface by different methods. In the literature, many studies explained the metal oxides coating mechanism for textiles i.e., pad-dry, hydrothermal, sol–gel, and chemical vapor deposition [23,24,25]. Our assumption is based on the fundamentals of nanotechnology, i.e., due to higher surface area and lower surface to volume ratio, nanoparticles show significantly higher performance than their bulk materials. Nanocoated textiles can provide significantly higher functional properties than microcoated materials. In recent years, researchers worked with the stabilization of nZnO on different substrates and reported their results. Maleki et al. examined photocatalytic degradation of humic substances by nZnO coated on glass plates. Chemical precipitation approach was used for the fabrication of nZnO [26]. In another study, Salehi et al. examined the effects of Cu-doped nZnO for the removal of RB-5 dye under sunlight. They used statistical tools to optimize process variables and design dye removal experiments [27]. These methods are time-consuming as all of them are multistep processes as well as an essential need of stabilizer is required to achieve small size of particles for these methods.

The benefits of ultrasonic energy in the preparation of nanomaterials have been proven as an economical, facile, non-toxic, and environmentally benign approach [28,29,30]. Many types of nanomaterials were coated on textile materials by sonochemical approach [31,32]. This method has shown its potential to augment washing stability and finishing applications. In present work, we have used ultrasonic energy for the fabrication of C-nZnO composites with significantly enhanced antimicrobial properties. The developed composites can be utilized for medical applications i.e., wound healing in hospitals and other places where there is a possibility of presence of bacteria. The UV screening action of nZnO is beneficial in enhancing the antimicrobial performance of C-nZnO composites as more UV absorption accelerates the photocatalytic mechanism of ZnO. The designed study involves an in situ fabrication and consequent attachment of nZnO on cotton under ultrasonic system. This work was conducted to investigate the role of ultrasonic horn intensity, ultrasonic irradiation time and precursor amount on the adhesion of nZnO on cotton and antimicrobial performance of C-nZnO composites. To the best of our knowledge, this study is unique in its scope as there is no relevant literature found for C-nZnO composites for antimicrobial performance in this manner. Ultrasonic probe homogenizer was used for this study. The work presented here is novel and this method can be used for the development of other types of composite materials.

## 2. Materials and Methods

### 2.1. Materials

A plain-woven bleached cotton fabric (100% pure) with 138 gm^−2^ areal density, 47 warp and 28 weft threads respectively were used for this study. Zinc chloride (ZnCl_2_), nitric acid (HNO_3_), sodium hydroxide (NaOH), ethylene diamine tetraacetic acid (EDTA) (C_10_H_16_N_2_O_8_), and ethanol (C_2_H_5_OH) were purchased from Sigma Aldrich (Prague, Czech Republic). All chemicals were of analytical grade and used without any further modification during the synthesis of C-nZnO composites.

### 2.2. In Situ Fabrication of C-nZnO composites

**Figure 1:**
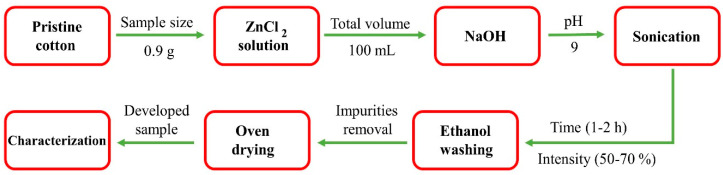
The proposed mechanism for the development of C-nZnO composites.

In a single step, nZnO with pure crystalline hexagonal phase were successfully synthesized and coated on cotton fabric (12 cm × 12 cm) with an average initial mass of 0.9 g. Initially, pristine cotton swatches were dipped in a 200 mL beaker containing 90 mL distilled water and then gradual addition of ZnCl_2_ was carried out. NaOH (1 M) was added in running solution to complete the reaction as well as to maintain pH (pH = 9) of the solution. The solution was then sonicated with high intensity ultrasonic system (Bandelin Sonopuls HD 3200, 20 kHz, 200 W, Berlin, Germany) for several time intervals under varying intensity based on our experimental design as shown in Table 1. The effective power of ultrasonic waves was 90 Wcm^−2^. During continuous sonication, the temperature of the system was increased until 70 °C, and in order to maintain temperature (40 °C), the system was placed in a cooling bath during the reaction. After sonication, the fabricated C-nZnO composite samples were washed with water and ethanol to remove all traces of impurities and further dried at 60 °C in an oven. It was observed that ultrasonic energy reduces size of nanoparticles and significantly increases crystallinity and fixation of nZnO with cotton fabric. The schematic visualization of proposed mechanism for C-nZnO composites is presented in Figure 1. Some initial experiments were performed in order to roughly estimate the optimal conditions for a successful study. We observed that prolonged sonication time and increased temperature show no adverse effect on color properties of samples. The deposited amount of nZnO on cotton (wt%) was calculated by Equation (1):

(1) $$Weight\;Percentage\;\left(wt\;\%\right)=\left[\frac{w_{s}-w_{b}}{w_{b}}\right]\times 100$$

where *w_s_* is the oven dry weight of sample after sonication and *w_b_* is the initial oven dry weight of pristine (untreated) sample.

### 2.3. Extraction of Solid Powder

After sample removal from the solution, the remaining solution was centrifuged for 5 min at 5000 rpm. The centrifuged solid (ZnO nanoparticles precipitated in the solution after sample removal) was washed several times with ethanol to eliminate impurities. This extraction was performed to detect crystal phase and particle size of nZnO.

### 2.4. Characterization of nZnO and C-nZnO Composites

Surface topography of C-nZnO composites and morphology of nZnO were studied through ultrahigh-resolution scanning electron microscopy (UHR-SEM) (Zeiss Ultra Plus, Carl Zeiss Meditec AG, Jena, Germany) with an accelerating voltage 2 kV equipped with an energy dispersive X-ray (EDX) spectrometer Oxford X-max 20 (Jena, Germany). Before analysis, the charging effect was neutralized by using local N_2_ injection. EDX analysis was performed at 10 kV. The X-ray diffraction pattern were collected by an X’Pert PRO X-ray diffractometer (manufactured by Malvern Panalytical Ltd., Malvern, UK,) using Cu Kα radiations with wavelength λ = 0.15406 nm at voltage 40 kV and current 30 mA. The scanning angle (2θ) range was from 5° to 80° with step size of 0.02° respectively. For a standard comparison and confirmation of pure wurtzite crystals of ZnO, collected patterns were matched with standard patterns of International Centre for Diffraction Data (ICDD) Powder Diffraction File (PDF: 89–7102). Scherrer’s crystallite equation (Equation (2)) was used to calculate crystal size:

(2) $$D=\frac{K\lambda }{\beta Cos\theta }$$

where *D* represents crystal size, *λ* shows wavelength, *β* represents full line width at half maximum height (FWHM), and *K* is shape constant having a constant value 0.89.

The deposited amount of nZnO on cotton was evaluated by volumetric titration with EDTA under inductively coupled plasma atomic emission spectroscopy (ICP-AES). For ICP-AES analysis, a PerkinElmer optima 2100 DV (SpectraLab Scientific Inc., Markham, ON, Canada) was used.

### 2.5. Antimicrobial Evaluation

Both qualitative and quantitative methods were used to evaluate antimicrobial performance of C-nZnO composites against *Staphylococcus aureus* (*S. aureus*) (ATCC 6538) and *Escherichia coli* (*E. coli*) (ATCC 25922) bacteria. In qualitative method, antimicrobial activity was evaluated by growth of inhibition (also known as disc diffusion) susceptibility method (Kirby–Bauer). In this method, a preincubated bacterial suspension was inoculated and grown in agar plates for 24 h at 37 °C to form a smooth layer of bacteria and then a reference sample (untreated or pristine cotton) with developed composites sample of 9 mm diameter were placed in agar dishes. The dishes were then incubated in a bacteriological oven for 12 h. After that, the halos were measured by ruler from the first zone of inhibition and the total width of inhibition of growth of bacteria given by diffusion halos was measured.

In a quantitative method, AATCC (100-2012) was followed to evaluate antimicrobial activity of C-nZnO composites by counting colonies forming units (CFU) of bacteria. Samples (9 mm of diameter) were placed in agar plates containing NaCl solution. Before counting, the initial optical density was adjusted as 0.1 at 600 nm. The initial bacterial concentration was (1.09 × 10^7^ CFU.mL^−1^). The bacterial culture was incubated for 12 h at 37 °C. The number of viable cells was recorded by counting bacteria colonies in agar plate before and after test and results were reported as percentages of bacteria cells reduction according to Equation (3).

(3) $$R\;\%=\left[\frac{\left(A-B\right)}{A}\right]\times 100$$

where *A* and *B* show total numbers of bacteria colonies recovered from untreated and nZnO coated samples and *R* shows reduction percentage.

### 2.6. Ultraviolet Protection Factor (UPF)

UPF measurements were performed according to joint Australian/New Zealand standard (AS/NZS 4399:2017) with Shimadzu Europa UV-3101PC spectrophotometer (Duisburg, Germany). Five measurements were conducted with different directions and their average was considered as final value of UPF. Equation (4) was used to calculate UPF value,

(4) $$UPF=\frac{\sum_{280}^{400}S_{\lambda }E_{\lambda }\Delta_{\lambda }}{\sum_{280}^{400}S_{\lambda }E_{\lambda }T_{\lambda }\Delta_{\lambda }}$$

where $S_{\lambda }$ is solar spectral irradiance, $E_{\lambda }$ is relative erythemal spectral response, $T_{\lambda }$ is average spectral transmittance, and $\Delta_{\lambda }$ is measured wavelength interval.

### 2.7. Leaching Durability and Washing Stability

The durability and stability of C-nZnO composites were determined against two different methods, i.e., leaching and washing. In leaching, the total number of Zn^+2^ ions existing in leaching solution was calculated and compared with the initial solution. NaCl (1M) solution was used as a reference solution and samples were leached at laboratory conditions. All samples were treated with NaCl solution at room temperature for 8 h and after that total amount of Zn^+2^ ions were estimated by ICP-AES analysis.

A washing test was performed according to standard test method ISO 105 C06 (B1M). The experiment was repeated for three consecutive washing cycles and for each cycle 4 gL^−1^ detergent was used at 50 °C. The sample was then rinsed and dried at 60 °C. The total reduction in weight (wt%) was calculate according to Equation (1).

### 2.8. Tensile Strength

A mechanical property (tensile strength) of the developed C-nZnO composites was tested on TIRA Test 2300 (TIRA GmbH, Schalkau, Germany) according to standard test method ISO 13934-1, under constant rate of extension.

## 3. Results and Discussion

### 3.1. SEM and EDX Analysis

**Figure 2:**
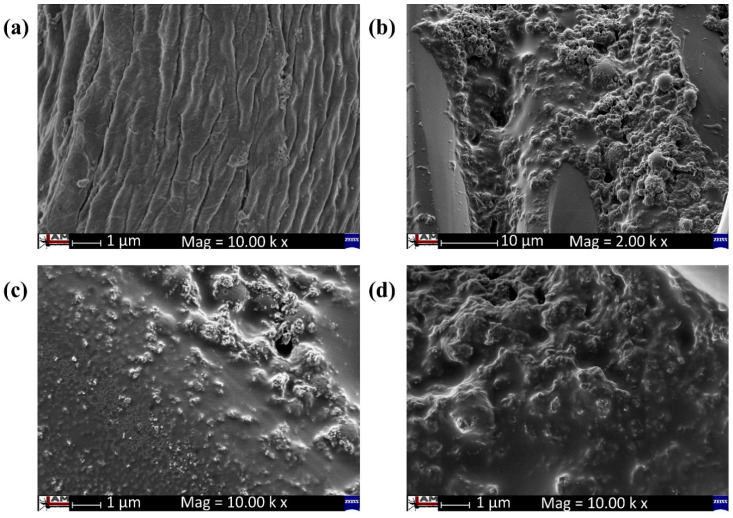
UHR-SEM analysis of (**a**) untreated sample, (**b**) sample C-nZnO 3, (**c**) sample C-nZnO 5, and (**d**) sample C-nZnO 7.

**Figure 3:**
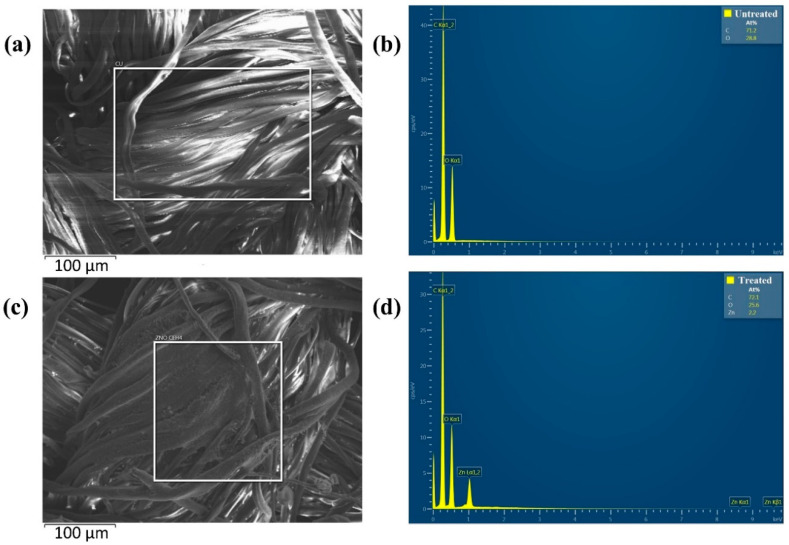
EDX analysis of (**a**) the untreated sample (the rectangular part shows a smooth and clear surface), (**b**) EDX spectrum of untreated sample, (**c**) sample C-nZnO 1 (the rectangular part shows the existence of nZnO as a thick condense layer on cotton, and (**d**) EDX spectrum of sample C-nZnO 1.

SEM micrographs and EDX spectra for the evaluation of surface topography of C-nZnO composites and morphology of synthesized nZnO before and after sonication were taken at 2.00 k and 10.00 k magnification. Figure 2a–d represents the results of SEM analysis, while Figure 3a–d shows the illustration of EDX analysis. Figure 2a shows the very clean and smooth surface of the untreated sample (pristine cotton). However, all other synthesized samples indicate the presence of nZnO (Figure 2b–d). Higher magnification was used to visually judge the presence and coating of nZnO on cotton. It can be observed that majority of particles were randomly distributed on cotton and covered the surface like a condensed thick layer. The observed shape of synthesized nanoparticles was quasi-spherical.

EDX analysis was performed between the untreated sample and sample C-nZnO 1 to detect elemental composition and weight percentage. Figure 3a,b demonstrates a clear EDX spectrum of pristine cotton as no elemental peak of Zn was found, whereas Figure 3c,d confirms the presence of Zn element on sample C-nZnO 1. The atomic percentage of Zn was high for all synthesized samples that indicates a higher loading of nZnO on cotton. The inset of Figure 3c was taken to show the homogenous coating of nZnO on cotton and to calculate particle size. The average particle size determined by ImageJ software from SEM images was 27.7 nm.

In sonication, the final product yield and particle size strongly depend on acoustic cavitation and reactants concentration. The selection of variables, i.e., concentration, time and intensity were based on the above-mentioned assumption. Finding out the optimized conditions for the selected variables was one of the aims of this study. We observed that increasing horn intensity gave a lower value of weight percentage (Table 1). We assume that augmentation of particles collision from a certain limit may cause detachment of particles from fabric surface. We also observed that an augmentation in reactants concentration and sonication time result in larger particle size. It happened because during continuous sonication, pressure, and rate of adsorption increased together that results in multilayer particles formation that enhance the clustering (agglomeration) of particles. This increases the particle size to some extent. Therefore, by a diminution in sonication time, horn intensity and reactants concentration, we have optimized the process and obtained optimal conditions for nZnO deposited amount, UPF, particle size, and antimicrobial performance. These results demonstrate the benefits of ultrasonic energy from application point of view in textiles as well as in material sciences. The observed EDX and SEM results are in agreement.

### 3.2. XRD Pattern

**Figure 4:**
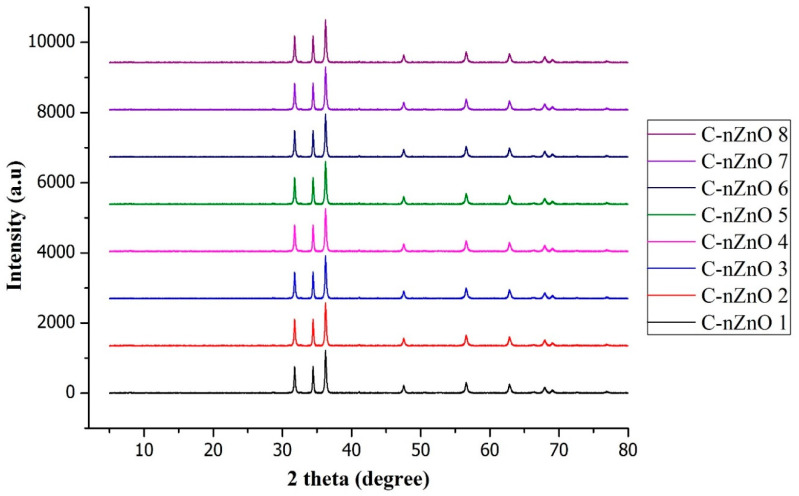
XRD diffraction patterns of all treated samples.

The crystal size, phase, and structure of nZnO were determined by XRD analysis. The collected patterns of extracted solid powders were compared with standard ICDD file (PDF: 89-7102) and presented in Figure 4. The results confirm that nZnO synthesized by sonication were crystalline in nature and formed hexagonal crystalline structure. The results demonstrate that all the sharp diffraction peaks matched with standard XRD diffraction patterns of ZnO. A series of crystalline peaks at 2θ = 31.7°, 34.4°, 36.2°, 47.5°, 56.6°, 62.8°, and 67.9° are assigned to [100], [002], [101], [102], [110], [103], and [112] planes, respectively. These plane reflections confirm the natural arrangement of hexagonal wurtzite nZnO. No traces of impurities, i.e., Zn(OH)_2_, were detected during XRD. For all samples, the average particle size estimated by equation 2 was 27.4 nm. However, the particle size for sample C-nZnO 1 was 26.8 nm (Table 1). The major effect of sonication was found on crystallization. Ultrasonic energy is responsible for crystallization process and played a crucial part in the synthesis process of nZnO. In our previous study, we explained that ultrasonic energy designs the crystal structure by modifying peaks intensity. During the synthesis process, ultrasonic waves enhances peaks intensity that results in more sharper peaks of pure crystals [30]. SEM and EDX results are in well agreement with XRD results.

### 3.3. ICP-AES Analysis

The ICP-AES analysis confirmed the presence of nZnO on cotton for all sonochemically treated samples, while the presence of elemental Zn was not found for untreated sample. For amount estimation, samples were first neutralized with 1M HNO_3_ solution for 2 h. The extracted solution for each sample was then titrated against EDTA and inserted into a nebulizer that converts the sample into a specific wavelength of metals that any sample contains. The characteristics elemental peak of Zn was detected in the spectrum and counted. ICP-AES results are mentioned in Table 1.

### 3.4. Sonochemical Synthesis and Deposition (Coating) of nZnO on Cotton

Using an in situ sonochemical method, nZnO were synthesized and coated on cotton by the following reactions.

(5) $$ZnCl_{2}+2NaOH\to Zn{\left(OH\right)}_{2}+2NaCl$$

(6) $$Zn{\left(OH\right)}_{2}\to ZnO+H_{2}O$$

Zn(OH)_2_ clusters were produced as an intermediate product during the hydrolysis process. These clusters were further dissolved into ions, i.e., Zn^+2^ and OH¯, and then converted into ZnO by dehydration of OH^¯^ during the final step of reaction mechanism. During the synthesis of nZnO, the already synthesized nanoparticles were pushed on towards the surface of cotton by pressure gradient resulting a physical adsorption of nZnO on cotton. Additionally, the phenomenon of acoustic cavitation worked as a local hot spot where pressure and temperature are at their extremely peak conditions. These conditions are responsible for the generation of free OH and H radicals [30]. These free unstable radicals promote the overall reaction and produce zinc oxide nanoparticles (nZnO). During the development of C-nZnO composites, acoustic cavitation phenomenon was performed among the liquid and inside the yarn and among the fabric and the boundary layer of liquid. During ultrasonic irradiations, fluid flow accelerates and, as a result, good adsorption of nZnO on the surface of cotton was found. We observed that during sonication, two different phenomena, i.e., acoustic cavitation and extremely high local heating conditions, occurred at same time, which not only enhanced the migration of nZnO on cotton surface, but also opened the fiber internal structure at the interface, resulting in a strong adherence of particles with the fabric surface.

### 3.5. UPF

The absorption of ultraviolet (UV) rays is a natural tendency of ZnO. The UV spectrum of solar radiations is classified as UVC with radiation range 100 to 280 nm, UVB with radiation range 280 to 320 nm, and UVA with radiation range 320 to 380 nm. UVC radiations are absorbed by ozone layer and only UVA and UVB radiations reach on earth surface. These radiations are highly reactive and cause sunburn, skin cancer, and other skin diseases. The antimicrobial performance of developed samples is based on photocatalytic reaction. More UV absorption increases the chances for photocatalytic mechanism. UPF rating directly evaluates UV radiations absorption efficiency of all developed samples. A high UPF rating means higher absorption of UV radiations and vice versa. The overall results of UPF rating are reported in Table 1. However, a comparison of previously performed studies with the present study is reported in Table 2. The UPF values varied from 2 (untreated sample) to 216 (sample C-nZnO 1). These results confirmed that UPF value mainly depends on the particle size as well as deposited amount.

### 3.6. Photooxidative Mechanism

**Figure 5:**
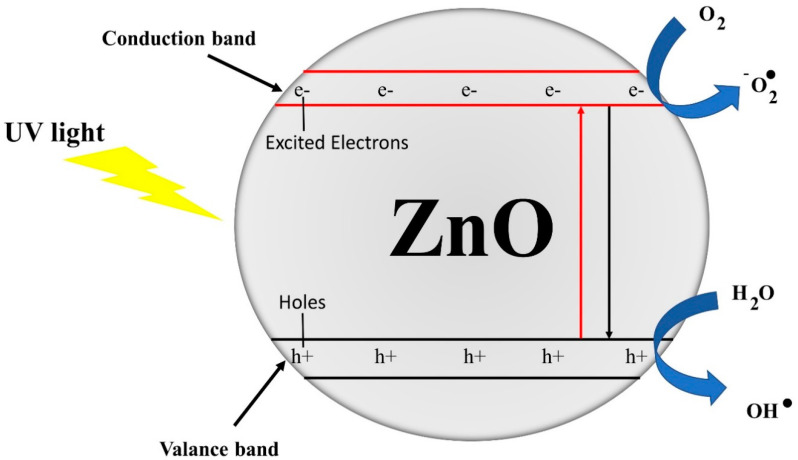
The proposed reaction mechanism on ZnO surface [29].

ZnO is considered a good photocatalyst at industrial scale. When light energy (photon with greater band gap energy than ZnO, i.e., 3.2 to 3.3 eV) strikes the surface of ZnO, electrons are released and combine with oxygen in the air and become super oxide anion (^●^O_2_^−^). Moreover, surface of ZnO becomes positively charged (creation of holes) and takes electron from moisture of air. The moisture that has lost electron becomes hydroxyl radical (OH^●^). These hydroxyl radicals (OH^●^) and superoxide anion (^●^O_2_^−^) are known as reactive oxygen species (ROS). These ROS, due to their strong power of oxidation, decompose organic compounds that cause bad smell and staining. Zhang et al. [36] explained that high production of (OH^●^) radicals on the surface accelerates decomposition power. The proposed photooxidative reaction mechanism on ZnO surface is presented in Figure 5.

### 3.7. Antimicrobial Activity

#### 3.7.1. Qualitative Method

The antimicrobial activity of C-nZnO composites was evaluated against *S. aureus* and *E. coli* by zone of inhibition test. The results did not show any zone for untreated sample. However, a clear zone of inhibition was observed for all C-nZnO composite samples. The results for all samples after 12 h of treatment are presented in Table 3. A significant zone was observed for all samples and sample C-nZnO 1 showed maximum results for inhibition zone i.e., 6.2 ± 0.3 for *S. aureus* and 5.9 ± 0.7 for *E. coli*, respectively.

#### 3.7.2. Quantitative Method

The results regarding quantitative antimicrobial test of all samples against *S. aureus* and *E. coli* were evaluated by counting the number of bacteria cell colonies after 12 h of incubation time. Again, the incubation of untreated sample did not show any significant change on bacteria cells viability. However, 100% reduction (*R%*) was observed against *S. aureus* and 98% reduction was observed against *E. coli* for sample C-nZnO 1. The overall results of antimicrobial activity (qualitative and quantitative) are reported in Table 3.

### 3.8. Durability against Leaching

The release of nanoparticles into the surrounding environment is considered one of the influential factors exploiting the commercialization of nanocoated bandages. Even though the main hazard in this study was the release of nZnO into washing affluent, we also investigated the content of Zn^+2^ ions in leaching solution. The estimation of Zn^+2^ ions is also important as zinc inhibits the growth of many microbes to some extent. The content of Zn^+2^ ions in the leaching solution was 15 ppm, 17 ppm, 24 ppm, 19 ppm, 49 ppm, 30 ppm, 70 ppm, and 47 ppm for sample C-nZnO 1 to sample C-nZnO 8, respectively. The results confirm that only 4.3% Zn^+2^ of ions were removed from C-nZnO 1 surface by leaching with NaCl solution. However, the percentage removal of Zn^+2^ ions for samples C-nZnO 2 to C-nZnO 8 was 6.0%, 5.4%, 4.6%, 5.7%, 4.9%, 5.1%, and 4.5%, respectively. The percentage reduction for all sonochemically synthesized C-nZnO composites is negligible from application point of view. Therefore, the leaching test results confirm that sonochemically deposited nZnO is strongly anchored to the cotton surface as a minimal amount was released even after 8 h leaching.

We observed that sample C-nZnO 1 had shown excellent results for antimicrobial activity, content of nZnO on cotton, particle size, UPF, and leaching. Therefore, further experiments, i.e., washing and reusability, were only conducted with this sample.

### 3.9. Washing Stability

**Figure 6:**
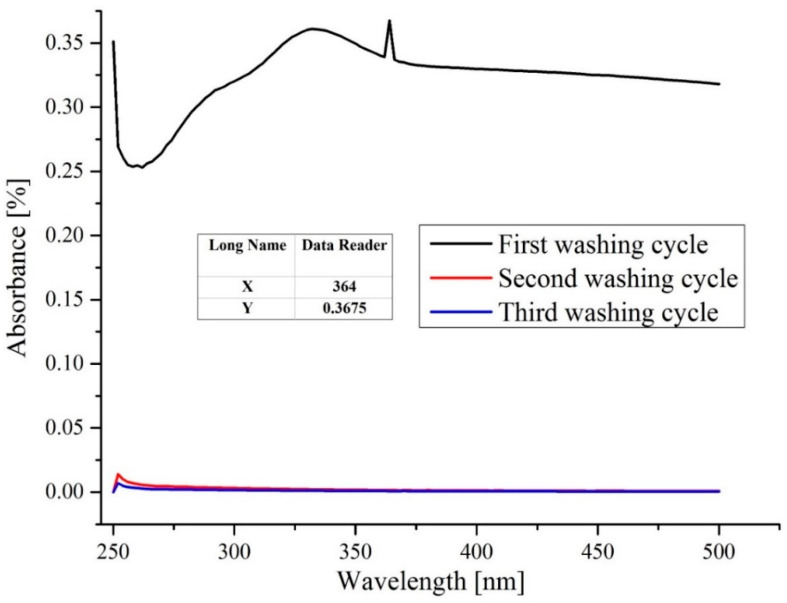
Absorbance spectra of C-nZnO 1 after different washing cycles.

Washing stability is a significantly important attribute of any nanostructured-based textile composite for commercialization. The washing stability of sample C-nZnO 1 was evaluated by repeating its application for three consecutive cycles. After each cycle, the sample was dried and reuse for next cycle. The released amount of nZnO in the washing bath was considered as durability against washing. The lower amount nZnO released indicates more stability and vice versa. The washing solution was analyzed by spectrophotometer. During the first washing cycle, as shown in Figure 6, an absorption peak at 364 nm (characteristic absorption peak of ZnO) was observed, indicating the existence of nZnO in solution. The inset shows the data reader peak reading. It occurred as some of the loosely attached and physically unstable nanoparticles were released from sample surface and migrated into washing bath during first wash. However, the results of the next cycles confirmed the absence of nZnO into washing bath as no peak was detected. These results confirm that sonochemically synthesized C-nZnO composites are very stable and an excellent choice for antimicrobial bandages.

**Figure 7:**
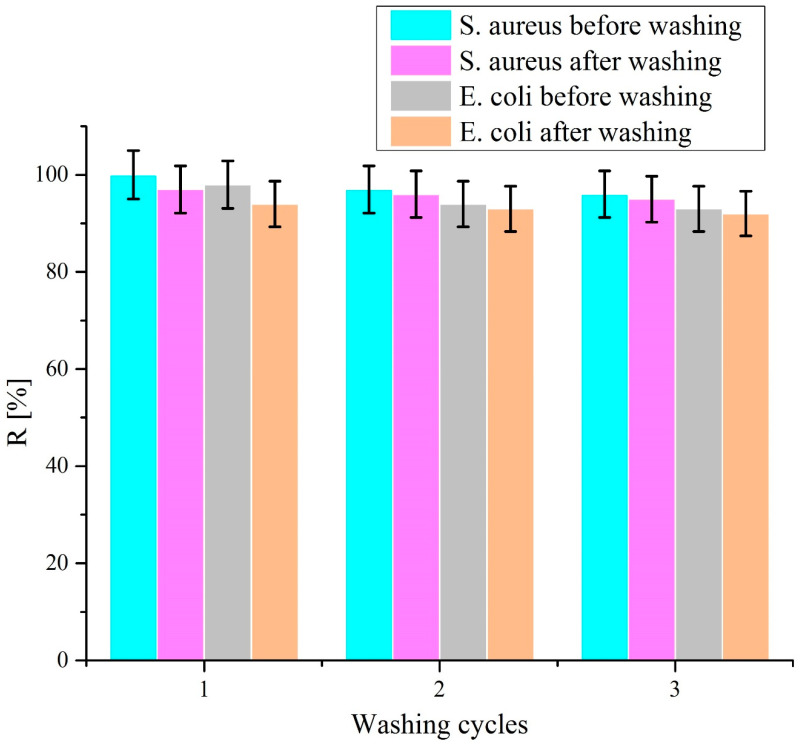
Reusability performance of C-nZnO 1 against *S. aureus* and *E. coli.*

**Figure 8:**
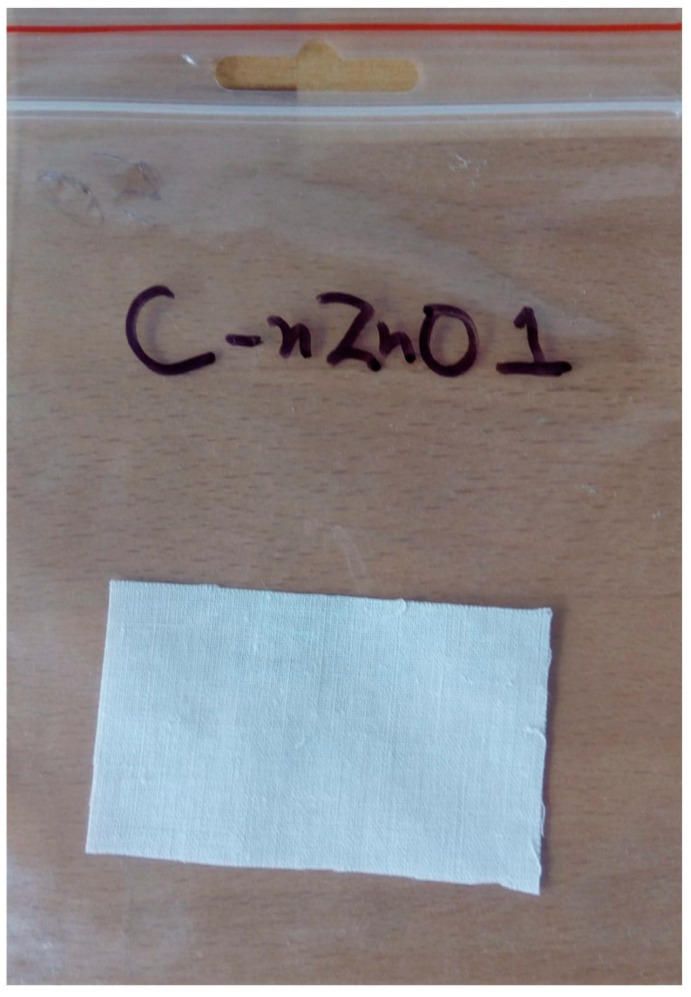
The real image of sample C-nZnO 1.

The reusability of the washed specimen (C-nZnO 1) was also checked against both microbes. The statistical significance of the data was performed at 95% confidence interval. For each cycle, sample C-nZnO 1 was placed in *S. aureus* and *E. coli* growth cultures for 4 h and reduction percentage (*R%*) of bacteria colonies were determined. *R*% was lost only 5% against *S. aureus* and 6.1% against *E. coli* after third wash as illustrated in Figure 7. This change is negligible and the obtained results confirm that developed C-nZnO composites are durable, stable, and reusable, and can be considered as an excellent choice for antimicrobial textiles. The real image of sample C-nZnO 1 is shown in Figure 8.

### 3.10. Tensile Strength of C-nZnO composites

The results of breaking force for the untreated sample and sample C-nZnO 1 were 531 N and 523 N with standard deviations of 1.9 and 2.2, respectively. The observed results were almost similar for untreated and treated samples, showing that ultrasonic irradiations and experimental conditions did not damage cotton fiber structure to a significant level.

## 4. Conclusions

C-nZnO composites were successfully synthesized and coated in a single step by sonication. The as-synthesized nZnO samples were thoroughly distributed and firmly anchored on cotton surface as confirmed by leaching and washing. XRD patterns confirmed the formation of pure crystals of ZnO that further explained the effects of ultrasonic irradiations during synthesis mechanism. The average particle size for nZnO calculated by mathematical equation was 27.4 nm applicable for functional applications especially medical applications, i.e., wound dressings and antimicrobial bandages. Ultrasonic irradiations and high loading of nZnO showed no negative effect on the structural and mechanical properties of cotton fabric. Moreover, composite sample C-nZnO 1 showed excellent results as an antimicrobial agent under optimized conditions.

## Figures and Tables

**Table 1:** Experimental detail and results of developed C-nZnO composites.

Sample Name	ZnCl_2_ [g]	Reaction Time [min]	Horn Intensity [%]	nZnO Deposited Amount [wt %]	nZnO Deposited Amount [ppm]	Particle Size [nm]	UPF
Untreated	-	60	50	-	-	-	2
C-nZnO 1	10	60	50	2.2	348	26.8	216
C-nZnO 2	10	60	70	1.7	283	27.2	189
C-nZnO 3	10	120	50	4.9	439	27.5	165
C-nZnO 4	10	120	70	4.3	405	27.3	112
C-nZnO 5	20	60	50	11.1	855	27.2	96
C-nZnO 6	20	60	70	7.8	608	28.2	74
C-nZnO 7	20	120	50	22.2	1372	27.3	101
C-nZnO 8	20	120	70	16.7	1025	28.1	76

**Table 2:** A comparative analysis of various ZnO coated cotton composites for UPF.

Sr. No.	Coating Method	UPF	Reference
1	Wet chemical	106	[33]
2	Two-step solvothermal	183	[34]
3	Hydrothermal	158	[35]
C-nZnO 1	Sonochemical	216	Present work
C-nZnO 2	Sonochemical	189	Present work
C-nZnO 3	Sonochemical	165	Present work

**Table 3:** Qualitative and quantitative antimicrobial tests for C-nZnO composites.

Sample Name	Qualitative Test		Quantitative Test	
	*S. aureus*	*E. coli*	*S. aureus*	*E. coli*
	Halos Diameter [mm]	Halos Diameter [mm]	Reduction Percentage [%]	Reduction Percentage [%]
C-nZnO 1	6.2 ± 0.3	5.9 ± 0.7	100	98
C-nZnO 2	5.4 ± 0.1	5.3 ± 0.4	99	98
C-nZnO 3	5.6 ± 0.6	5.4 ± 0.5	98	97
C-nZnO 4	5.1 ± 0.1	4.7 ± 0.1	98	97
C-nZnO 5	5.5 ± 0.5	5.7 ± 0.3	100	98
C-nZnO 6	4.8 ± 0.3	4.4 ± 0.2	98	96
C-nZnO 7	4.1 ± 0.4	3.9 ± 0.5	97	96
C-nZnO 8	3.4 ± 0.6	3.1 ± 0.8	97	96
